# Co‐occurrence of breast cancer and neuroendocrine tumours: New genetic insights beyond Multiple Endocrine Neoplasia syndromes

**DOI:** 10.1002/edm2.92

**Published:** 2019-09-08

**Authors:** Vincent Larouche, Amit Akirov, Emily Thain, Raymond H. Kim, Shereen Ezzat

**Affiliations:** ^1^ Division of Endocrinology and Metabolism Department of Medicine University Health Network University of Toronto Toronto Ontario Canada; ^2^ Division of Endocrinology and Metabolism Jewish General Hospital McGill University Montreal Quebec Canada; ^3^ Familial Cancer Clinic Princess Margaret Cancer Centre University Health Network Toronto Ontario Canada; ^4^ Fred A Litwin Family Centre in Genetic Medicine University Health Network Toronto Ontario Canada; ^5^ Division of Medical Oncology Department of Medicine University of Toronto Toronto Ontario Canada

**Keywords:** breast neoplasm, carcinoid tumour, endocrinology, medical genetics, medical oncology, Multiple Endocrine Neoplasia, mutation, neuroendocrine tumours

## Abstract

**Objective:**

Age‐standardized incidence of female breast cancer is 145.1 per 100000/year and 5.86 per 100000/year for neuroendocrine tumours (NET) in Canada. Evidence is scarce about gene variants that may predispose patients to develop both neoplasms. The objective of this study was to identify germline gene variants associated with this combination of tumours.

**Design and patients:**

A retrospective chart review (2007‐2018) in a tertiary NET referral centre was completed. A series of 9 female patients with concurrent breast cancer and NET is presented. All patients underwent a 37 gene hereditary cancer next‐generation sequencing panel.

**Results:**

Mean age was 61.4 years (35‐85) at breast cancer diagnosis and 63.4 years (51‐89) at NET diagnosis. Four patients had a pancreatic, three had a small bowel and two had a lung NET. Two patients were known cases of *MEN1,* and one patient was found to harbour a pathogenic variant in *MEN1* and a variant of unknown significance (VUS) in *ATM*. A second patient was found to harbour a pathogenic variant in *APC*. A third patient was found to carry a pathogenic variant in *PALB2* as well as a VUS in *FANCM, MLH1* and *STK11*. Another patient was found to harbour a VUS in *MSH2*. One patient was found to carry a pathogenic variant in *NTHL1*.

**Conclusion:**

The first cases of a *PALB2*, an *APC* and a *NTHL1* pathogenic variants in patients with both breast cancer and NET were presented. NGS testing should be considered in specific patients with this combination of neoplasms, as certain germline variants beyond *MEN1*, have important implications for cancer surveillance.

## 1 INTRODUCTION

Age‐standardized incidence of female breast cancer was 145.1 per 100000/year in 2012^1^ in Ontario, Canada. Neuroendocrine tumours (NET) are less common, with an incidence of 5.86 per 100000/year in 2009 in the same population.^2^ Evidence is scarce about the possible germline (inherited) genetic variants that may predispose patients with NET to develop breast cancer or *vice versa*. Multiple Endocrine Neoplasia type 1 (MEN1) syndrome,^3^, ^4^, ^5^, ^6^ Multiple Endocrine Neoplasia type 4 (MEN4, caused by *CDKN1B*)^7^, ^8^ and Cowden syndrome (*PTEN*)^9^ have been associated with both NET and breast cancer in case reports or case series. A whole‐genome sequencing study of 102 pancreatic NET patients by Scarpa et al reported novel pathogenic mutations in DNA repair genes *MUTYH, CHEK2* and *BRCA2*, which are well‐known breast cancer susceptibility genes.^10^ However, NET are not incorporated into genetic testing guidelines for breast cancer patients.^11^ Recently, a SEER database retrospective study^12^ showed that the incidence of pancreatic NET is higher in patients with a first primary cancer of the pancreas, bladder, thymus and female breast cancer. However, multiple institutional cohort studies have reported a higher incidence of gastrointestinal and genitourinary second primary malignancies in patients with NETs^13^, ^14^, ^15^ and an Italian national database study^16^ of patients with a primary diagnosis of bronchopulmonary NET has shown a higher incidence of thyroid neoplasms in women and a higher incidence of kidney and bladder tumours in men. Importantly, these studies did not show a higher incidence of breast cancer in NET patients. We here report a case series of nine female patients with a concurrent diagnosis of breast cancer and NET (lung, GI tract or pancreatic primary) evaluated at a tertiary NET referral centre between 2007 and 2018 as well as the results of a 37 gene next‐generation sequencing panel that was performed on all patients. To our knowledge, this is the first study using this approach to report on possible gene variants linking the co‐occurrence of breast cancer and NET.

## 2 METHODS

Three hundred and fifty‐three patients with a NET (lung, pancreas or gastrointestinal) were evaluated at our tertiary NET referral centre between 2007 and 2018 (Princess Margaret Cancer Centre, Toronto, Ontario, Canada). Of these, 206 were female of whom 21 were diagnosed with breast cancer either prior to NET diagnosis or during follow‐up. Nine patients accepted to take part in the study and are detailed in this report. Four were lost to follow‐up prior to the accrual period and eight declined to participate in the study. The breast cancer could have been diagnosed prior to the NET diagnosis, synchronously (here defined as within 6 months before of after the NET diagnosis) or metachronously (more than 6 months after the NET diagnosis). Patients' data including clinical data (age, sex, age at diagnosis of each tumour and follow‐up time for each tumour), breast cancer data (affected breast, surgical intervention, hormone receptor status, surgical pathology reports, imaging reports, treatments including chemotherapy, radiation therapy, aromatase inhibitor therapy, trastuzumab and other therapies) were recorded from the electronic medical record and clinic chart. Given that our NET clinic is a provincial tertiary referral centre, some patients had their breast cancer treated in another hospital; therefore, some details on the breast cancer data were unavailable. Patient's NET data including primary site, surgical intervention, WHO grade, surgical pathology reports, imaging reports, treatments including somatostatin analogue therapy, mTor inhibitor therapy (everolimus), peptide receptor radionuclide therapy (PRRT with 177‐Lutetium), capecitabine‐temozolomide chemotherapy and other therapies, presence and location of metastases and treatment modalities of metastases were recorded from the electronic medical record. All patients underwent a 37 gene next‐generation sequencing (NGS) panel including the following genes: *APC, ATM, BARD1, BMPR1A, BRCA1, BRCA2, BRIP1, CDH1, CDK4, CDKN2A‐P14, CDKN2A‐P16, CHEK2, CTNNA1, EPCAM, FANCC, FANCM, FLCN, GREM1, HOXB13, MEN1, MLH1, MSH2, MSH3, MSH6, MUTYH, NBN, NTHL1, PALB2, PMS2, POLD1, POLE, PTEN, RAD51C, RAD51D, SDHB, SMAD4, STK11,* and *TP53* as part of standard of care investigations. All patients signed written consent to be included in this case series, and our institution's research ethics board approved the study.

## 3 RESULTS

### 3.1 Clinical cases: Breast cancer data

Breast cancer data are summarized in Table 1 for all study participants. All patients (n = 9) were female, with a mean age of 61.4 years (35‐85) at breast cancer diagnosis. With regards to surgical resection, six patients had a lumpectomy, two had a mastectomy, and one was not a surgical candidate due to her advanced age. In terms of adjuvant therapy, two patients received chemotherapy, four received radiotherapy, six received an aromatase inhibitor and one received trastuzumab. Surgical pathology revealed that five tumours were invasive ductal carcinomas, one was a ductal carcinoma in situ, and two were lobular carcinomas. Mean tumour size was 1.1 cm (0.4‐2.0). Three tumours had a histological grade 2 and one had a grade 3. All tumours were Stage 1a. Hormone receptor status for tumours was the following: seven tumours were ER+, 5 were PR + and only one was HER‐2/Neu+. Mean follow‐up time for the breast cancer was 7.44 years (2‐26) Three patients had locoregional recurrence. Two patients developed breast cancer metastases; one in cervical lymph nodes and bone metastases and one had liver metastases. Four patients had their breast cancer diagnosed before their NET, 2 patients had both malignancies diagnosed synchronously (defined here as within 6 months) and three patients were diagnosed with breast cancer metachronously (ie more than 6 months after NET diagnosis).

**Table 1:** Clinical and pathological characteristics of patients' breast neoplasm

ID	Age at breast CA Dx	Affected breast	Surgery type	Histology	ER	PR	HER‐2/Neu	Maximal size (cm)	Histological Grade	Stage	Presence and location of metastases	CTx	RTx	AI	Other therapy	Follow‐up Time Breast CA (y)
1	35	Left	Lumpectomy	NA	+	+	‐	NA	NA	NA	Left brachial plexus recurrence 2010, Liver mets 2018	No	Yes	Yes	NA	26
2	57	Right	Lumpectomy	Invasive ductal carcinoma	‐	‐	+	1.2	3	T1cN0M0	Mediastinal LN, Cervical LN, bone mets, Recurrence right breast 2016 (Triple negative)	Yes	Yes	No	Herceptin	2
3	56	Right	Mastectomy	Invasive ductal carcinoma	+	+	‐	0.4	NA	T1aN0M0	No	No	No	No	No	11
4	72	Right	Lumpectomy	Invasive ductal carcinoma	+	‐	‐	0.5	2	T1aNxMx	Local recurrence 2017	No	No	Yes	NA	2
5	62	Right	Lumpectomy	Invasive ductal carcinoma	+	+	‐	1.3	NA	NA	No	Yes	Yes	Yes	NA	8
6	62	Right	Lumpectomy	Ductal carcinoma in situ	NA	NA	NA	NA	NA	NA	No	No	No	No	No	3
7	85	Right	Not surgical candidate	Invasive ductal carcinoma	+	‐	‐	NA	2	NA	No	No	No	Yes	NA	5
8	48	Right	Mastectomy	Lobular carcinoma	+	+	‐	NA	2	T1N0M0	No	No	No	Yes	No	6
9	76	Left	Lumpectomy	Invasive lobular carcinoma	+	+	‐	2.0	NA	T1cN0M0	No	No	Yes	Yes	No	4

Abbreviations: AI, Aromatase inhibitor therapy; CTx, Chemotherapy; ER, Oestrogen receptor; LN, Lymph nodes; NA, Not available; PR, Progesterone receptor; RTx, Radiation therapy.

### 3.2 Clinical cases: Neuroendocrine tumour data

Neuroendocrine tumours data are summarized in Table 2 for all study participants. Of the nine patients, four had a pancreatic NET, three had a small bowel NET and two had a lung NET. Five patients had surgical resection of the primary tumour, three were not surgical candidates either due to age or metastatic disease and one was followed by active surveillance. Six patients were treated with a somatostatin analogue and four with everolimus. Two received capecitabine‐temozolomide chemotherapy, which targets both malignancies, and one received peptide receptor radionuclide therapy (PRRT with 177‐Lutetium). Mean tumour size was 5.2 cm (1.9‐8.1); one patient had Stage 1, two had Stage 2 and one had Stage three disease. One pancreatic NET was an insulinoma. Mean Ki‐67 index was 12.6% (1.0‐33.0). One NET had a WHO Grade 1 tumour, six had a Grade 2 and one had a Grade 3. Mean NET follow‐up time was 5.67 years (1‐10) Five tumours developed metastases during follow‐up, five to the liver, two to mesenteric lymph nodes, two to bones. Two patients had hepatic metastasectomy. Two patients were known cases of Multiple Endocrine Neoplasia type 1 (MEN1), one was known to harbour a pathogenic *MEN1* variant.

**Table 2:** Clinical and pathological characteristics of patients' neuroendocrine tumour

ID	Age at NET Dx	NET Site	Surgical resection	Maximal size (cm)	Staging	KI‐67 (%)	Mitotic count (per HPF)	WHOGrade	SSA	Everolimus	CAP‐TEM	PRRT	Presence and location of metastases	Therapy for metastases	NET Follow‐up time (y)	Other info
1	57	Duodenal NET	Not surgical candidate	NA	NA	3.0	NA	2	No	No	No	No	No	No	4	
2	53	PancreasNET	Distal pancreas + splenectomy	5.8	NA	20.0	1/50	2	Yes	Yes	Yes	No	Right liver + Right retroperitoneal nodule + Bone, pancreatic tail	Liver lobectomy + IVC, Right adrenal, retroperitoneal node resection	8	
3	57	PancreasNET	Active surveillance	NA	NA	NA	NA	NA	No	No	No	No	No	No	10	MEN‐1
4	65	Lung NET	RML + RLL resection	7.1	pT2N0M0	5.02	NA	2	Yes	Yes	No	Yes	Liver + Bone	No	9	MEN‐1
5	68	Small bowel NET	Not surgical candidate	NA	NA	33.0	8/10	3	Yes	No	Yes	No	Mesenteric LN + liver	No	2	
6	56	PancreasNET	Distal pancreas + splenectomy	2.5	pT2N0M0	1.0	NA	1	No	No	No	No	No	No	9	
7	89	Duodenal NET	Not surgical candidate	NA	NA	15.5	NA	2	Yes	Yes	No	No	Liver	No	1	
8	51	Lung NET	RML resection	1.9	pT1aN0M0	3.46	11/50	2	Yes	No	No	No	No	No	3	
9	75	PancreasNET	Distal pancreas	8.5	pT3N1M0	20.0	2/10	2	Yes	Yes	No	TBA	Liver + Mesenteric LN	Yes, liver segment 7 resection + mesenteric and coeliac nodes	5	

Abbreviations: CAP‐TEM, Capecitabine‐temozolomide chemotherapy; F‐U, Follow‐up;HPF, High power field; LN, Lymph node; MC, Mitotic count; NA, Not available; NET, Neuroendocrine tumour; PRRT, Peptide radionuclide radiation therapy; RLL, Right lower lobe; RML, Right middle lobe; SSA, Somatostatin analogue therapy.

### 3.3 Clinical Cases: Comprehensive hereditary cancer gene variant panel results

Results of the 37 NGS gene panel are summarized in Table 3 for all study participants.

**Table 3:** Results of comprehensive hereditary cancer gene panel

Patient ID	Gene Variants	Clinical Significance
1	No pathogenic variants detected	NA
2	No pathogenic variants detected	NA
3	*MEN1*: c.628_631delACAG, p.(Thr210Serfs*213)	Pathogenic
	*ATM*: c.483G > C, p.(GIn161His)	Unknown significance
4	*APC*: c.3920 T > A, p.(IIe1307Lys)	Likely pathogenic with low penetrance
5	*PALB2*: c.3549C > A, p.(Tyr1183*)	Pathogenic
	*FANCM*: c.3586C > T, p.(Arg1196Cys).	Unknown significance
	*MLH1*: c.‐7C > T	Unknown significance
	*STK11:* c.316C > T, p.(Arg106Trp)	Unknown significance
6	No pathogenic variants detected	NA
7	No pathogenic variants detected	NA
8	*MSH2:* c.965G > T, p. (Gly322Val)	Unknown significance
9	*NTHL1:* c.268C > T, p.(GIn90*)	Pathogenic

Patient 3 was diagnosed with a right breast invasive ductal carcinoma and underwent a radical mastectomy at age 56. She was synchronously diagnosed with nonfunctional pancreatic NET that are <2 cm in diameter and are being followed by imaging through an active surveillance protocol. She was a known case of Multiple Endocrine Neoplasia Type 1 (MEN1) with a confirmed *MEN1* variant before gene panel testing. She is known for a microprolactinoma managed medically on dopamine agonist medications, primary hyperparathyroidism for which she underwent parathyroidectomy, multiple nonfunctional pancreatic NET, and she had a right adrenalectomy for ACTH‐independent (adrenal) Cushing syndrome. Gene panel testing confirmed that she harbours a pathogenic variant in exon 3 of *MEN1* (c.628_631delACAG, p. Thr210Serfs*213). This patient was also found to harbour a variant of unknown significance in exon 5 *ATM [Ataxia‐Telangectasia Mutated]* (c.483G > C, p.GIn161His).

Patient 4 was diagnosed with a lung NET at age 65 and was treated successively with surgical resection, somatostatin analogue therapy, everolimus and peptide radionuclide radiation therapy (PRRT with 177‐Lutetium). She was metachronously diagnosed with a right breast invasive ductal carcinoma at age 72 and underwent lumpectomy and adjuvant therapy with an aromatase inhibitor. Although she has no known *MEN1* pathogenic variant, she was clinically considered to have MEN1^17^ with a history of hyperparathyroidism due to multiglandular hyperplasia for which she underwent parathyroidectomy and the lung NET, as described above.^18^ She was found to harbour a likely pathogenic variant in *APC [Adenomatous Polyposis Coli]* (c.3920T > A, p.IIe1307Lys). This variant has been associated with a modestly increased risk for developing breast and colorectal cancer (OR 1.5‐2.0)^19^ and is considered to be a low‐penetrance breast cancer susceptibility allele.^20^ It classified as a likely pathogenic variant with relatively low penetrance. This is the first report of this pathogenic *APC* variant in a case of breast cancer and lung NET.

Patient 5 was diagnosed with a right breast invasive ductal carcinoma at age 65 and underwent lumpectomy, adjuvant chemotherapy, radiation therapy and aromatase inhibitor therapy. This diagnosis preceded her small bowel NET for which she was managed with somatostatin analogue therapy and combination capecitabine + temozolomide chemotherapy. She was found to harbour a pathogenic variant in *PALB2* [*Partner and Localizer of BRCA2]* (c.3549C > A, p.Tyr1183*). This variant has been reported as a pathogenic variant in individuals with breast cancer and Fanconi anaemia.^21^, ^22^, ^23^ Similarly, a recent case report^24^ described a patient with metastatic pancreatic neuroendocrine carcinoma with a rare germline pathogenic frameshift variant in exon 5 of the *PALB2* gene denoted as c.2325dupA, p.Phe776Ilefs*26. Therefore, patient 5 in the current series with a right breast invasive ductal carcinoma and a small bowel NET represents a novel clinical association with this *PALB2* gene variant*.* Importantly, had she not taken part in this study, this patient would not have fulfilled genetic testing criteria based on her breast cancer presentation and family history. This patient was also found to harbour three variants of uncertain significance in *FANCM [Fanconi Anemia Complementation Group M]* (c.3586C > T, p.Arg1196Cys),^25^, ^26^ *MLH1 [MutL Homolog 1] MLH1*:c.‐7C > T,^27^ *STK11* [*Serine/Threonine Kinase 11*] (c.316C > T, p.Arg106Trp).

Patient 9 was diagnosed with an insulin‐secreting pancreatic NET (insulinoma) at age 75 and was managed with a distal pancreatectomy, somatostatin analogue therapy and everolimus. She was synchronously diagnosed with a left breast invasive lobular carcinoma and underwent lumpectomy, adjuvant radiation therapy and aromatase inhibitor therapy. She was found to harbour a single pathogenic variant in *NTHL1 [Nth Like DNA Glycosylase 1]* (c.268C > T, p.GIn90*). *NTHL1*‐associated polyposis is an autosomal recessive condition where individuals harbouring biallelic variants in *NTHL1* have been reported with a variety of different cancers.^28^, ^29^, ^30^, ^31^, ^32^ This is the first case of this same pathogenic variant being observed in a patient with a pancreatic NET and breast cancer.

Patient 8 was diagnosed with a right breast lobular carcinoma at age 48 and was managed with radical mastectomy and adjuvant aromatase inhibitor therapy. This preceded her diagnosis of lung NET at age 51, for which she was treated with surgical resection followed by somatostatin analogue therapy. She was found to harbour a VUS in *MSH2 [MutS Homolog 2]* (c.965G > T, p.Gly322Val).

Patients 1, 2, 6 and 7 had no variants detected from gene panel testing.

## 4 DISCUSSION

In this study, we report 9 cases of female patients with co‐occurrence of breast cancer and a NET (lung, GI tract or pancreas primary) evaluated at a tertiary NET referral centre between 2007 and 2018. During this period, 21 out of 206 female NET patients evaluated at our centre had a concurrent diagnosis of breast cancer; Eight declined to participate in the study and four were lost to follow‐up prior to the accrual period. This is in‐line with the 1/9 frequency of breast cancer in the general female population. The aim of our study was not to demonstrate that female NET patients have a higher incidence of breast cancer, but rather to illustrate that the genetic mutational landscape of patients with a combination of NET and breast cancer may differ from that of female breast cancer in the general population. All patients underwent a 37 NGS gene panel as part of standard of care investigation, which revealed one patient with a pathogenic variant in *MEN1*, one patient with a pathogenic *PALB2* variant*,* one patient with a likely pathogenic variant in *APC* and another patient with a pathogenic variant in *NTHL1*. Five VUS were also identified in *ATM, FANCM, MLH1, STK11* and *MSH2*. Given the pervasiveness of gene panel testing in breast cancer patients, our findings suggest that gene panel testing should be conducted on patients with a combination of breast cancer and NET regardless of family history.

At this point, only case reports or series have illustrated the increased risk of having combined breast cancer and NET in MEN1,^3^, ^4^, ^5^, ^6^ MEN4^7^, ^8^ and Cowden syndrome.^9^ Germline variants in *MUTYH, CHEK2* and *BRCA2*, well‐known breast cancer susceptibility genes were also identified in a whole‐genome sequencing study of 102 pancreatic NET patients.^10^ Although one recent SEER national database retrospective study showed an increased risk of pancreatic NET in patients with another first primary malignancy including female breast cancer, this was not found in other population studies.^13^, ^14^, ^15^, ^16^

Here, we report likely pathogenic and pathogenic variants in *APC* and *PALB2* in cases of co‐occurrence of breast cancer and NET. These pathogenic variants are respectively known to be associated with breast and colorectal carcinoma (*APC*), breast cancer and Fanconi anaemia for *PALB2.* The National Comprehensive Care Network (NCCN) Guidelines recommend patients with *PALB2* variants undergo high‐risk screening with breast MRI and mammography.^11^ Similarly, the NCCN recommends patients who harbour the APC p.Ile1307Lys variant should undergo colonoscopy every 5 years starting at the age of 40.^33^, ^34^ We also report one patient with a single pathogenic variant in *NTHL1*. No specific NCCN Guidelines currently exist for patients harbouring single *NTHL1* pathogenic variants, but the spectrum of this disorder is only being fully delineated.^35^ Despite evidence for *MEN1* pathogenic variants being associated with breast cancer and neuroendocrine tumours, current Endocrine Society Clinical Practice Guidelines suggest only regular biochemical and imaging (CT or MRI) investigations for detection of pituitary adenomas, parathyroid adenomas and hyperplasia, bronchial and thymic NET, gastroenteropancreatic NET and adrenal neoplasms for MEN1 patients with no mention of breast or colorectal cancer screening.^17^ All patients who harbour a germline gene variant should have genetic counselling. Family members of those with likely pathogenic or pathogenic variants should be identified and offered testing.

Identification of certain variants in genes such as *PALB2* or *APC* have important implications for cancer surveillance in patients and their at‐risk relatives. Although multiple primary tumours is a well‐known indication for genetic testing, this is not addressed by any guidelines in the breast cancer or NET literature. Interestingly, had participants not taken part in our study, they would not have qualified for NGS testing as per current breast cancer guidelines. Our findings illustrate that germline cancer gene panel testing should be considered in specific patients with a co‐occurrence of breast cancer and NET and these panels should include MEN1 and other hereditary cancer syndrome genes.

Our study presents several limitations. First, the small number of cases (n = 9) and limited number of genes analysed in this comprehensive hereditary cancer gene variant panel that the patients underwent hinders the generalizability of our findings to other populations and the claim of a causal relationship between all identified variants, whether pathogenic or of unknown significance, and cases of co‐occurring female breast cancer and NET. Tumour testing examining for second hits or loss of heterozygosity may shed light on the role of these variants in the NET and breast cancers. Larger studies are required, possibly enrolling patients from national databases, and completing whole‐genome sequencing on such patients to identify a wider array of potentially associated genes.

## 5 CONCLUSION

Patients with one malignancy are more often predisposed to develop a second neoplasm, prompting clinicians to consider this possibility when new lesions appear during follow‐up. This case series described the role of gene panel sequencing in specific patients with a combination of breast cancer and NET. As confirmed in one of our study patients, pathogenic variants in the *MEN1* gene are associated with a higher incidence of breast cancer and NET. Beyond Multiple Endocrine Neoplasia syndromes, however, we report the first cases of patients with a *PALB2*, an *APC* and a *NTHL1* pathogenic variants and both malignancies. Germline cancer gene panel testing should be considered in individual patients with a co‐occurrence of breast cancer and NET, as identification of certain gene variants would have important implications for cancer surveillance in patients and their at‐risk relatives. Further studies are needed to clarify the genetic drivers of this unique combination of endocrine with nonendocrine neoplasia.

## CONFLICTS OF INTEREST

The authors have no conflict of interest to disclose.

## AUTHORS’ CONTRIBUTIONS

Author Vincent Larouche was the principal investigator of the study. He completed the Ethics Board application, contacted patients, organized patient visits and blood draws, collected data and prepared the manuscript. Author Amit Akirov helped with the literature review, data collection and manuscript preparation. Author Emily Thain is a Genetics Counsellor who helped with literature review, manuscript preparation and proceeded to genetic counselling of all patients who underwent NGS testing. Author Raymond Kim is a Medical Geneticist who contributed to the literature review and manuscript preparation and oversaw the genetic testing of subjects. Author Shereen Ezzat is an Endocrine Oncologist, and he was the main supervisor for the study.

## ETHICS STATEMENT

All patients signed written consent to be included in this case series, and our institution's research ethics board approved the study. (University Health Network, Toronto, ON, Canada).

## Data Availability

The data that support the findings of this study are available on request from the corresponding author. The data are not publicly available due to privacy or ethical restrictions.
